# Redescription of the rare amphipod crustacean *Pseudaeginella montoucheti* (Quitete, 1971) from Brazil

**DOI:** 10.3897/zookeys.146.1856

**Published:** 2011-11-09

**Authors:** Mariana Baptista Lacerda, Ichiro Takeuchi, Setuko Masunari

**Affiliations:** 1 Postgraduate Program in Zoology, Federal University of Paraná. Centro Politécnico, Jardim das Américas, Caixa Postal 19020, CEP 81.531–970 – Curitiba, Paraná, Brazil; 2 Department of Life Environment Conservation, Faculty of Agriculture, Ehime University.3–5-7 Tarumi, Matsuyama 790–8566, Japan

**Keywords:** Amphipoda, *Pseudaeginella montoucheti* (Quitete 1971), Redescription, Santa Catarina, Brazil

## Abstract

*Pseudaeginella montoucheti* (Quitete, 1971) is redescribed based on newly collected specimens from red and brown algae and tubiculous polychaete colony that were obtained from shallow waters at Tamboretes Archipelago, Balneário Barra do Sul and Sepultura Beach, Bombinhas, Santa Catarina State, Brazil. Of 10 species of *Pseudaeginella* so far reported, *Pseudaeginella montoucheti* is closest to *Pseudaeginella sanctipauli* Laubitz, 1995*,* but differs from the latter by having more numerous body spines including ventro-lateral ones over gills on pereonites 3 and 4, and the antenna 1 length measuring half body length. An identification key for *Pseudaeginella* species and a checklist of Caprellidea occurring along the Brazilian coasts are also presented.

## Introduction

The knowledge on ecology and biology of Brazilian caprellids is restricted to some areas and substrata, mainly those living on macroalgae from southeastern and southern coast (Masunari 1982; Dutra 1988; Arenzon and Bond-Buckup 1991; Wakabara et al. 1991; Dubiaski-Silva and Masunari 1995; Mittmann and Müller 1998; Jacobucci et al. 2002; Jacobucci et al. 2006; Leite et al. 2007; Dubiaski-Silva and Masunari 2008). In the same way, taxonomic studies about Brazilian caprellids are also limited to few descriptions of new species (McCain 1968; Quitete 1971a, b, 1972; Serejo 1998; Guerra-García 2003a; Rayol and Serejo 2003) and two redescriptions (Serejo 1997; Masunari and Takeuchi 2006).

Of 19 species of caprellids so far recorded from Brazilian coasts (Table 1), *Pseudaeginella montoucheti* was firstly described as *Fallotritella montoucheti* Quitete, 1971 (Quitete 1971a),based on specimens collected from Pernambuco State, northeastern Brazil. In the middle of 1990's, Laubitz (1995) synonymed the genus *Fallotritella* with the genus *Pseudaeginella*, leading to change subsequently into *Pseudaeginella montoucheti* (Quitete, 1971). The present study redescribes *Pseudaeginella montoucheti* based on individuals from Santa Catarina State, southern Brazil with the special reference to characteristics of mouthpart and appendages. Additionally, a key for the species of the genus *Pseudaeginella* and a checklist of Caprellidea occurring along the Brazilian coasts are also presented.

**Table 1.** Checklist of the Brazilian Caprellidae with their distribution in Brazil

**Species**	**Distribution in Brazil (references)**
**Caprellidae*Caprella* Lamarck, 1801**	
*Caprella aculeata* (Dana, 1853)	Rio de Janeiro (Wakabara and Serejo 1998)
*Caprella andreae* Mayer, 1890	Torres and Tramandaí, RS (Arenzon and Bond-Buckup 1991)
*Caprella danilevskii* Czerniavski, 1868	Ubatuba, SP (Jacobucci et al. 2002; Cunha et al. 2008), Paranaguá, PR (Dutra 1988), Matinhos, PR (Dubiaski-Silva and Masunari 1995), Bombinhas, SC (Dubiaski-Silva and Masunari 2008)
*Caprella dilatata* Krøyer, 1843	Arraial do Cabo, RJ (Serejo 1998), Ubatuba, SP (Jacobucci et al. 2002), Santos, SP (Jacobi 1987), Peruíbe, SP (Jacobucci et al. 2006), Penha, SC (Masunari and Takeuchi 2006)
*Caprella equilibra* Say, 1818	Ubatuba, SP (Jacobucci et al. 2002; Cunha et al. 2008), São Sebastião, SP (Flynn and Valério-Berardo 2009), Peruíbe, SP (Jacobucci et al. 2006), Bombinhas, SC (Dubiaski-Silva and Masunari 2008)
*Caprella globiceps* Dana, 1853	Rio de Janeiro (Wakabara and Serejo 1998)
*Caprella penantis* Leach, 1814	Paranaguá, PR (Dutra 1988), Matinhos, PR (Dubiaski-Silva and Masunari 1995), Bombinhas, SC (Dubiaski-Silva and Masunari 2008), Governador Celso Ramos, SC (Mittmann and Müller 1998)
*Caprella scaura* Templeton, 1836	Arraial do Cabo, RJ (Serejo 1998), Ubatuba, SP (Jacobucci et al. 2002; Leite et al. 2007; Cunha et al. 2008), São Sebastião, SP (Flynn and Valério-Berardo 2009), Peruíbe, SP (Jacobucci et al. 2006), Bombinhas, SC (Dubiaski-Silva and Masunari 2008)
***Hemiaegina* Mayer, 1890**	
*Hemiaegina minuta* Mayer, 1890 (= *Hemiaegina costai* Quitete, 1972)	Bahia and Pernambuco States (Quitete 1972; Serejo 1997)
***Liropus* Mayer, 1890**	
*Liropus nelsonae* Guerra-García, 2003	7°58'S, 34°17'W – 7°50'S, 34°17'W (Guerra-García 2003a)
***Monoliropus* Mayer, 1903**	
*Monoliropus enodis* Rayol & Serejo, 2003	Guanabara Bay, RJ (Rayol and Serejo 2003)
***Orthoprotella* Mayer, 1903**	
*Orthoprotella melloi* Quitete, 1975	Pernambuco State (Quitete 1975)
***Paracaprella* Mayer, 1890**	
*Paracaprella digitimanus* Quitete, 1971	1°21'S, 43°50'W (Quitete 1971b)
*Paracaprella pusilla* Mayer, 1890	Arraial do Cabo, RJ (Serejo 1998), Ubatuba, SP (Leite et al. 2007), São Sebastião, SP (Flynn and Valério-Berardo 2009)
*Paracaprella tenuis* Mayer, 1903	Ubatuba SP (Cunha et al. 2008), Bombinhas, SC (Dubiaski-Silva and Masunari 2008)
***Parvipalpus* Mayer, 1890**	
*Parvipalpus colemani* Guerra-García, 2003	7°58'S, 34°17'W – 7°50'S, 34°17'W (Guerra-García 2003a)
***Pseudaeginella* Mayer, 1890***Pseudaeginella montoucheti* (Quitete, 1971)	Itamaracá, PE, Mar Grande and Olivença, BA, Guarapari and Vitória, ES (Quitete 1971a), Arraial do Cabo, RJ (Serejo 1998), Ubatuba, SP (Jacobucci et al. 2002; Cunha et al. 2008), Pássaros Island (26°22'S, 48°31'W) and Araras Island (26°27'S, 48°34'W), Balneário Barra do Sul, SC (present study)
**Phtisicidae*Phtisica* Slabber, 1769**	
*Phtisica marina* Slabber, 1769	Arraial do Cabo, RJ (Serejo 1998), Peruíbe, SP (Jacobucci et al. 2006)
*Phtisica verae* Quitete, 1979	Rio de Janeiro State (Quitete 1979)

BA, Bahia State; ES, Espírito Santo State; PE, Pernambuco State; RJ, Rio de Janeiro State; SC, Santa Catarina State; SP, São Paulo State

## Materials and methods

Collections were conducted at two Islands - Pássaros Island (26°22'S, 48°31'W) and Araras Island (26°27'S, 48°34'W) – in Tamboretes Archipelago, municipality of Balneário Barra do Sul (16^th^ May 2009) and at Sepultura Beach, Bombinhas (30^th^ June 2011) Santa Catarina State, southern Brazil. Caprellideans were found in the phytal of the red algae *Amphiroa beauvoisii* Lamouroux and *Spyridia aculeata* (Schimper) Kützing, of the brown alga *Sargassum cymosum* C. Agardh, 1820 and as the associate fauna of a tubiculous polychaete colony; these communities were living over rocky surface in infralittoral depths, from 0.5 to 7.0 m. The biological substrates were carefully wrapped up in a plastic bag and scraped from the rocky surface with a spatula by scuba divers. In laboratory, the plastic bag content was very kindly washed in dilute formalin. The deposited material was sieved, sorted and caprellids were fixed and preserved in ethyl alcohol 70%.

From a total of 54 examined specimens (31 males and 23 females), several specimens of male and female were selected to be dissected under stereomicroscope. The dissected material was mounted in polyvinyl lactophenol. All figures were drawn with the aid of a *camera lucida*. Specimens are deposited in Museum of Natural History of Capão da Imbuia (MHNCI) and in Center for Zoological Studies (CEZ), from Institute of Biology, Federal University of Rio de Janeiro.

## Results

**Family Caprellidae Leach, 1814**

**Genus *Pseudaeginella* Mayer, 1890**

### Pseudaeginella montoucheti

(Quitete, 1971)

http://species-id.net/wiki/Pseudaeginella_montoucheti

**Figure 1.**
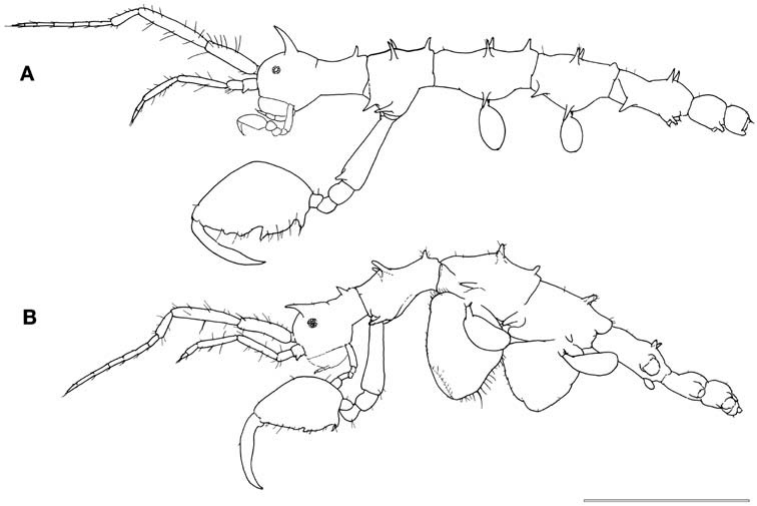
*Pseudaeginella montoucheti* (Quitete, 1971). **A** male, lateral view **B** female, lateral view. Scale bar: 1.0 mm.

Figs 1 [Fig F2] [Fig F3] –4

**Figure 2. F2:**
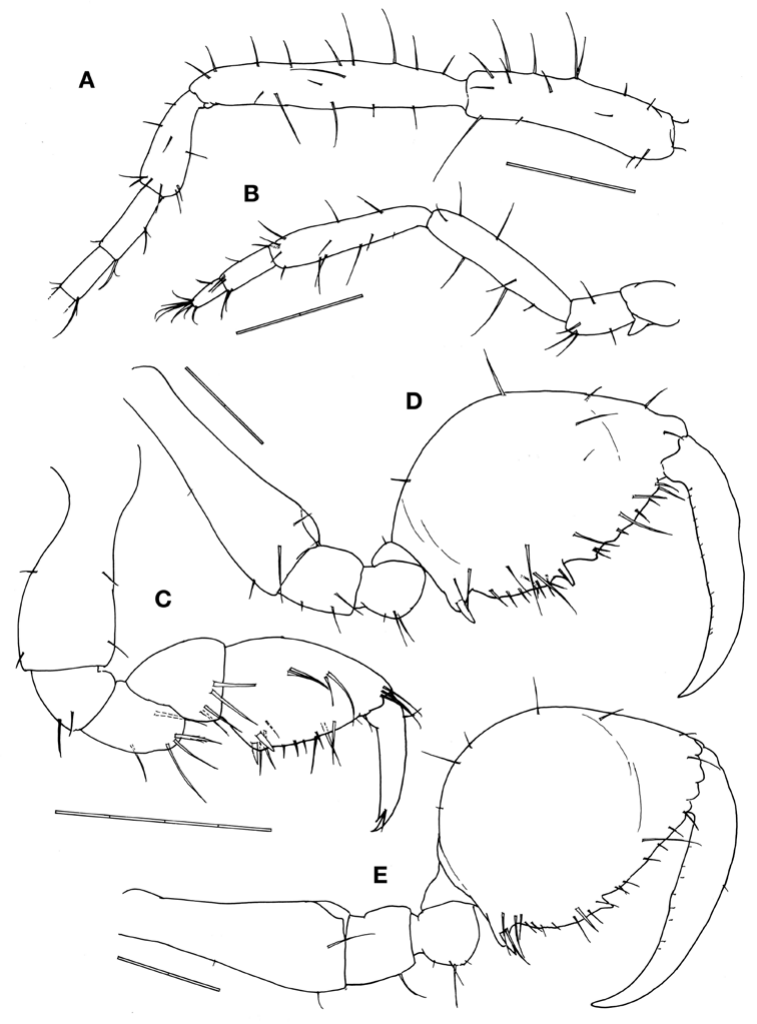
*Pseudaeginella montoucheti* (Quitete, 1971). **A–D** male. **A** antenna 1 **B** antenna 2 **C** gnathopod 1 **D** gnathopod 2. **E** female gnathopod 2. Scale bars: **A–E:** 0.2 mm.

Fallotritella montoucheti : Quitete 1971a, p.189–192, figs. 1–2
Pseudaeginella montoucheti – Laubitz, 1995, p.88.

#### Material examined.

MHNCI 2844 One female from the phytal of red alga *Spyridia aculeata*, 7 m deep, Araras Island (26°27'S, 48°34'W), Tamboretes Archipelago, Santa Catarina, Brazil, 16^th ^May, 2009.

MHNCI 2845 One male and two females from the phytal of the calcareous red alga *Amphiroa beauvoisii*, 4 m deep.

MHNCI 2846 Three males and two females from the phytal of *Amphiroa beauvoisii*, 1.5 m deep, Pássaros Island (26°22'S, 48°31'W), Tamboretes Archipelago, Santa Catarina, Brazil, 16th May, 2009.

MHNCI 2847 Four males and two females from the phytal of brown alga *Sargassum cymosum*, Bombinhas (27°08'28"S, 48°28'42"W), Santa Catarina, Brazil, 30^th^ June, 2011.

CEZ 968 Holotype male from of *Sargassum,* Itamaracá, Pernambuco, Brazil 5^th^ August, 1968. Collector: Dr. Pierre Montouchet.

CEZ 971 Two paratypes males and three paratypes females from of *Sargassum*, Mar Grande, Bahia, Brazil, 22th January, 1968. Collector: Dr. Pierre Montouchet.

CEZ 972 12 paratypes males and eight paratypes females from of *Sargassum*, Itamaracá, Pernambuco, Brazil 5^th^ August, 1968. Collector: Dr. Pierre Montouchet.

CEZ 973 Seven paratypes males and four paratypes females from of *Sargassum*, Mar Grande, Bahia, Brazil, 22th January, 1968. Collector: Dr. Pierre Montouchet.

CEZ 974 One paratype male and one paratype female from of *Sargassum*, Guarapari, Espírito Santo, Brazil, 6^th^ September, 1968. Collector: Dr. Pierre Montouchet.

Male (Fig. 1A). Body length 3.0 mm. Pereonites 3 and 4 the longest, followed by pereonites 2 and 5. Head and pereonite 1 (suture clearly present) concave along dorsal margin, head with an anteriorly curved mid-dorsal projection, pereonite 1 with a small postero-dorsal projection. Pereonite 2with paired mid-dorsal projections, 1 postero-dorsal projection, paired antero-lateral projections and paired mid-lateral projections. Pereonite 3 with paired mid-dorsal projections, 1 postero-dorsal projection and paired mid-lateral projections. Pereonite 4 with paired mid-dorsal projections, 1 weak postero-dorsal projection, paired antero-lateral projections and paired mid-lateral projections. Pereonite 5 withpaired mid-dorsal projections, paired antero-lateral projections and paired mid-lateral projection near the swollen basal part of pereopod 5. Pereonite 6 with paired postero-lateral projections near the basal part of pereopod 6.

Antennae (Figs. 2A, 2B). Antenna 1 about half body length. Peduncular articles with ca. 10 to 20 simple setae of varied length; peduncular article 2 the longest followed by article 1. Flagellum 6-articulate with 4/5 of peduncular length. Antenna 2 about 4/5 of antenna 1 length, without swimming setae; peduncular setose in varied length; flagellum with 8 and 6 simple setae in the proximal and distal articles.

**Figure 3. F3:**
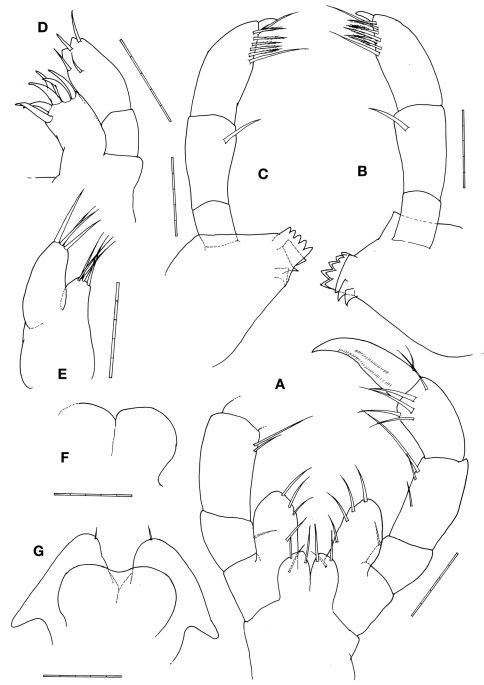
*Pseudaeginella montoucheti* (Quitete, 1971). Male. **A** maxilliped **B** left mandible **C** right mandible **D** maxilla 1 **E** maxilla 2 **F** upper lip **G** lower lip. Scale bars: **A–G:** 0.05 mm.

Mouthparts (Figs. 3A–G). Upper lip notched, forming rounded projections. Right mandiblewith incisor with 5 teeth and followed by lacinia mobilis with 5 teeth and 3 trapezoid plates; palp article 2 with 1 lateral seta; palp article 3 setal formula 1–6–1 with a distal knob. Left mandible incisor with 5 teeth followed by 3 trapezoid plates; palp article 2 with 1 lateral seta; palp article 3 setal formula 1–6–1 with a distal knob. Lower inner lips round and fused each other, outer lobes round with 1 apical seta. Maxilla 1outer plate with 6 stout apical setal-teeth; palp distal margin with 4 setae. Maxilla 2inner plate triangular with 4 apical setae; outer plate elongate with about 4 apical setae. Maxilliped basal endite (inner plate) with 2 setae on outer margin; ischial endite (outer plate) oval, 2 times longer than inner plate, with 4 or 5 setae on inner margin; palp article 2 with 2 or 3 setae on inner margin; palp article 3 with 5 distal setae; palp article 4 (dactylus) weakly falcate.

Gnathopod 1 basis as long as ischium, merus and carpus combined, covered by sparse setae of varied length; propodus subtriangular, palm with a pair of proximal stout setae (grasping spines) and a row of 8 simple setae; dactylus with sparse and short setae, inner margin smooth with a teeth subdistally (Fig. 2C).

Gnathopod 2 inserted in the pereonite 2 at 2/5 from anterior margin (Fig. 1A); coxa vestigial; basis 1.3 times of pereonite 2 length, with a spiny projection near antero-distal corner; ischium rectangular; merus rounded; carpus triangular and provided with scarce simple setae; propodus oval, ratio between width: length = 0.57, inner margin provided with 1 stout setae proximally, 3 triangular projections medially and distally and numerous setae: few simple setae on the outer margin; dactylus shorter than palm and slightly curved with a row of setulae alongside the inner margin (Fig. 2D).

**Figure 4.**
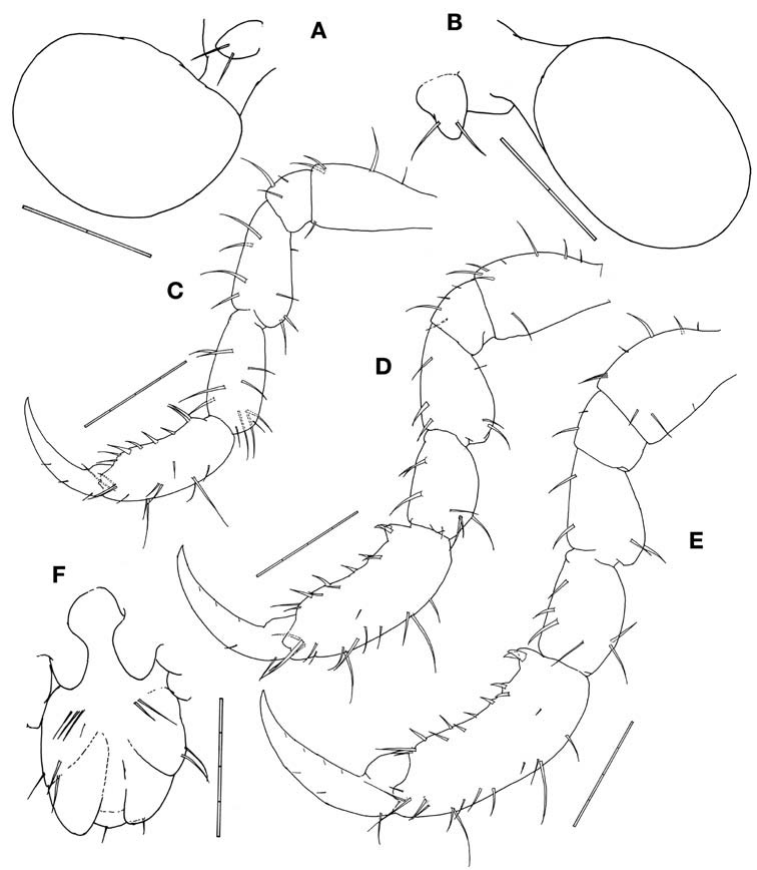
*Pseudaeginella montoucheti* (Quitete, 1971). Male. **A** pereopod 3 and gill 3 **B** pereopod 4 and gill 4 **C** pereopod 5 **D** pereopod 6 **E** pereopod 7 **F** abdomen (ventral view). Scale bars: **A,B**: 0.1 mm. **C–F**: 0.2 mm.

Gill 3length 2/5 of corresponding pereonite, elliptical (Fig. 1A), pereopod 3 tiny with 2 simple setae apically (Fig. 4A). Gill 4length 1/3 of corresponding pereonite, elliptical (Fig. 1A), pereopod 4 similar to pereopod 3 (Fig. 4B).

Pereopod 5 basis to carpus furnished with 3–10 setae of varied length; palm of propodus very slightly concave with 2 setae proximally and a row of 7 robust setae alongside; dactylus slightly curved (Fig. 4C). Pereopods 6 and 7 similar to pereopod 5 in feature but increasing in size (Figs. 4D, 4E).

Penes length about 2 times width (Fig. 4F).

Abdomen with a pair of lateral lobes and dorsal lobe with a pair of dorsal setae (Fig. 4F).

Female. Body length 3.1 mm (Fig. 1B). Pereonites 3 and 4 subequal and the longest, followed by pereonite 2. Clear suture between head and pereonite 1, head with 1 anteriorly curved mid-dorsal projection. Antenna 1 flagellum 7-articulate. Pereonite 1with 1 postero-dorsal projection. Pereonite 2with paired mid-dorsal projections, 1 postero-dorsal projection and paired antero-lateral projections. Pereonite 3with paired mid-dorsal projections, 1 postero-dorsal projection, paired mid-lateral projections and paired postero-lateral projections. Pereonite 4with paired mid-dorsal projections and paired mid-lateral projections. Pereonite 5with paired mid-dorsal projections. Gnathopod 2 propodus length 1.5 times width (Fig. 2E), with grasping spine proximally followed by a serrated margin; two smooth triangular projections medially.

#### Intraspecific variation.

In adult males and females including those collected by Quitete (Quitete, 1971a) in Pernambuco State, the number of articles in the flagellum of antenna 1 varies from 5 to 7 during growth. The size reduction of the mid-dorsal projections on pereonites 3 and 5 mentioned by this author was only found among specimens studied by her. Setal formula for terminal article of mandibular palp can be 1–5-1 or 1–6-1. The body spination is rather constant among individuals summing up 30 spines in males.

#### Type locality.

Itamaracá, Pernambuco State, Brazil.

#### Distribution.

Western South Atlantic. Brazil. Itamaracá, Pernambuco State; Olivença, Ilhéus, Bahia State; Vitória and Guarapari, Espírito Santo State (Quitete 1971a). Ubatuba, São Paulo State (23°32'S, 45°10'W – 23°30'S, 45°08'W) (Jacobucci et al. 2002, 2009). Paranaguá Bay, Paraná State (25°31'S, 48 °30'W) (Neves 2006). Tamboretes Archipelago: Pássaros Island (26°22'S, 48°31'W) and Araras Island (26°27'S, 48°34'W), Balneário Barra do Sul and Bombinhas Beach, Bombinhas, Santa Catarina State (present study).

#### Habitats.

Amongst thallii of the brown seaweed *Sargassum* sp. (Quitete 1971a and present paper), *Sargassum cymosum* (Jacobucci et al. 2002) and *Sargassum filipendula* (Jacobucci et al. 2009); on boat hulls and floating piers (Neves 2006); amongst thallii of the red algae *Amphiroa beauvoisii* and *Spyridia aculeata* and tubular branches of polychaete colony (present paper).

#### Remarks.

Takeuchi (1993) proposed a classification with four families, Caprellidae, Caprogammaridae, Paracercopidae, and Phtisicidae for the Amphipoda Caprellidea (Cyamidae excluded) based on the cladistic analysis. *Fallotritella* and *Pseudaeginella* were set under the Caprellidae. At the same time, Laubitz (1993) proposed a classification with eight families, Caprellidae, Caprellinoididae, Caprogammaridae, Cyamidae, Paracercopidae, Pariambidae, Phtisicidae and Protellidae. In her classification system, *Fallotritella* and *Pseudaeginella* were included in Caprellinoididae which is considered to be more related to Paracercopidae and Phtisicidae than to Caprellidae.

The above treatment of *Fallotritella* and *Pseudaeginella* performed by Takeuchi (1993) was followed by Myers and Lowry (2003) and Vassilenko (2006). In the higher classification system of Myers and Lowry (2003) based on cladistic analysis of corophiid amphipods, Caprellidae, Caprogammaridae and Cyamidae are included among the Caprelloidea. The Caprellidaeof Myers and Lowry (2003) is composed of two subfamilies, Caprellinae and Phtisicidae. *Fallotritella* and *Pseudaeginella* with ca. 50 genera constitute the Caprellinae under the Caprellidae (Myers and Lowry 2003). Vassilenko (2006) reviewed the recent studies dealing her support to Takeuchi's (1993) treatment more than to Laubitz (1993) concerning to the phylogeny of the Caprellidea.

The genus *Fallotritella* was established based on *Fallotritella biscaynensis* McCain, 1968 collected from Florida, U.S.A, Antigua & Barbuda and St. Lucia (McCain 1968), just prior to Quitete (1971a). The suggestion of synonymy of these two genus, i.e., *Pseudaeginella* and *Fallotritella* was mentioned under remarks of *Pseudaeginella* by McCain (1968). The lack of reference materials of *Pseudaeginella tristanensis* (Stebbing, 1888), the type species of *Pseudaeginella*, has been led to the presumption towards absence of pereopods 3 and 4 in *Pseudaeginella*. At the same time, *Fallotritella biscaynensis* was recorded to possess 1-articulate pereopods 3 and 4 in the generic description for *Fallotritella*. He also noted that, in case of presence of pereopods 3 and 4 in the two known species of *Pseudaeginella*, *Fallotritella* would fall as junior synonym of *Pseudaeginella* (see McCain 1968, p. 100). Almost 30 years later, Laubitz (1995) examined individuals of *Pseudaeginella tristanensis* collected from Amsterdam Islands in the southern Indian Ocean and reported the synonymy of these two genera based on the presence of minute pereopods 3 and 4 on these specimens.

*Pseudaeginella montoucheti* (Quitete, 1971) is a tiny caprellidean that measures less than 3.5 mm in body length (see Fig. 1). Within this genus, *Pseudaeginella montoucheti* is the second spiniest species (total of 30 spines on the head and pereonites 1–7 of males) and only surpassed by *Pseudaeginella sanctipauli* that has a total of 33 spines on body surface. In the drawing of *Pseudaeginella montoucheti* from Pernambuco State performed by Quitete (1971a) the following body projections are missing in male: a pair of dorsal projections instead of one dorsal spine medially on pereonite 5 and ventro-lateral projections over the insertion of pereopod 5 and 6 on pereonite 5 and 6, respectively. In spite of the wide distribution, *Pseudaeginella montoucheti* showed a relatively low intraspecific variation in its external morphology.

Although restricted to the Atlantic coast of Brazil, the present study showed that *Pseudaeginella montoucheti* is distributed along more than 2,600 km, from tropical (Itamaracá Island, Pernambuco State, 7°44'S, 34°49'W) to subtropical (municipalities of Barra do Sul, 26°27'S, 48°34'W and Bombinhas, 27°08'S, 48°28'W, Santa Catarina State) latitudes. Recently, *Caprella dilatata* Krøyer, 1843 was also reported showing wide distribution from Sao Paulo State, Brazil (Jacobucci et al. 2002) to Mar del Cobo and Mar del Plata Harbour, Argentina along the south Atlantic coast of South America (Masunari and Takeuchi 2006).

*Pseudaeginella* Mayer, 1890 is currently composed of 10 species: *Pseudaeginella antiguae* Barnard, 1932 from Antigua and Barbuda, *Pseudaeginella biscaynensis* (McCain, 1968) from Florida, U.S.A., *Pseudaeginella campbellensis* Guerra-García, 2003b from subantarctic islands of New Zealand, *Pseudaeginella colombiensis* Guerra-García, Krapp-Schickel & Müller, 2006 from Colombia, *Pseudaeginella inae* Krapp-Schickel & Guerra-García, 2005 from Indonesia, *Pseudaeginella montoucheti* (Quitete, 1971) from Brazil, *Pseudaeginella polynesica* (Müller, 1990) from Bora Bora and Moorea, French Polynesia, *Pseudaeginella sanctipauli* Laubitz, 1995 from St. Paul and Amsterdam Islands, France, *Pseudaeginella tristanensis* (Stebbing, 1888) from Tristan da Cunha, and *Pseudaeginella vaderi* Guerra-García, 2004 from Australia.

Of 10 species of *Pseudaeginella*, the closest species to *Pseudaeginella montoucheti* can be considered *Pseudaeginella sanctipauli* that wasdescribed from St. Paul and Amsterdam Islands, South Indian Ocean (Laubitz 1995), since both are the spiniest species within the genus. On the other hand, *Pseudaeginella montoucheti* can be distinguished from *Pseudaeginella sanctipauli* by the spinier body, antenna 1 length equals half body in males, and presence of ventro-lateral spines over gills on pereonites 3 and 4.

A key to the species of *Pseudaeginella* is presented below; it was mainly based on the characteristics of body somites because these can be observed without dissections of mouthparts.

**Key to species of the genus *Pseudaeginella***

**Table:** 

1a	Antenna 1 longer than half of body length	2
1b	Antenna 1 equal or shorter than half of body length	5
2a	Basis of gnathopod 2 longer than propodus length	*Pseudaeginella sanctipauli* (Fig. 5A)
2b	Basis of gnathopod 2 shorter than propodus length	3
3a	Basis of gnathopod 2 approximately the length of pereonite 2	*Pseudaeginella biscaynensis* (Fig. 5B)
3b	Basis of gnathopod 2 longer than pereonite 2 length	4
4a	Pereonites 2, 3, 4 and 5 with lateral projections near the insertion of gnathopod 2, gills and pereopods 5	*Pseudaeginella colombiensis* (Fig. 5C)
4b	Pereonites 2, 3, 4 and 5 without lateral projections	*Pseudaeginella polynesica*(Fig. 5D)
5a	Pereonites with dorsal projections	6
5b	Pereonites without any dorsal projections	8
6a	Basis of gnathopod 2 provided with a rounded projection proximally	*Pseudaeginella campbellensis* (Fig. 5E)
6b	Basis of gnathopod 2 without any projection	7
7a	Pereonites 4 and 5 with a paired antero-lateral projections, body somites with a total of more than 30 projections	*Pseudaeginella montoucheti* (Fig. 6A)
7b	Pereonites 4 and 5 without any antero-lateral projections, body somites with a total of less than 30 projections	*Pseudaeginella tristanensis* (Fig. 6B)
8a	Basal article of antenna 2 peduncle with a distal projection, well marked suture between head and pereonite 1	*Pseudaeginella vaderi* (Fig. 6C)
8b	Basal article of antenna 2 peduncle without any projection, discrete suture between head and pereonite 1	*Pseudaeginella inae* (Fig. 6D)

**Figure 5.**
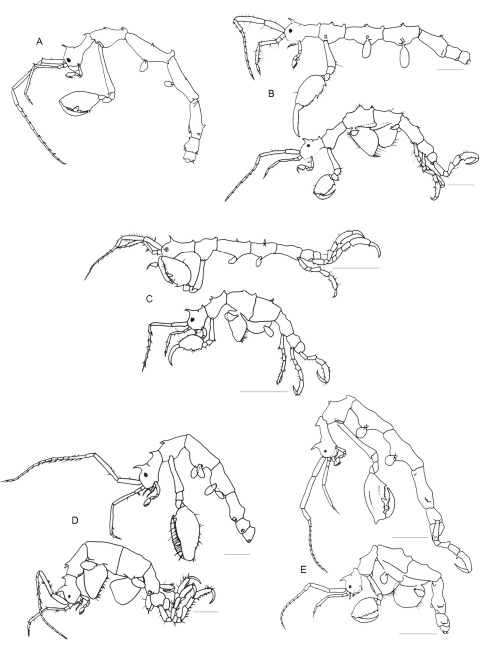
*Pseudaeginella* spp**. A** *Pseudaeginella sanctipauli* Laubitz, 1995 (Redraw from Laubitz 1995) **B** *Pseudaeginella biscaynensis* (McCain, 1968)(Redraw from Guerra-García 2002. Scale bar 0.05 mm) **C** *Pseudaeginella colombiensis* Guerra-García, Krapp-Schickel & Müller, 2006 (Redraw from Guerra-Garcíaet et al. 2006. Scale bar 1 mm) **D** *Pseudaeginella polynesica* (Müller, 1990. Scale bar 500 µm) (Redraw from Müller 1990) **E** *Pseudaeginella campbellensis* Guerra-García, 2003 (Redraw from Guerra-García 2003. Scale bar 1 mm).

**Figure 6.**
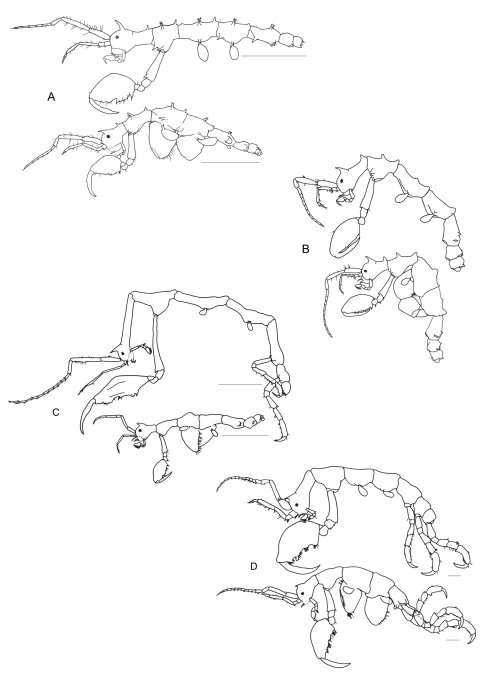
*Pseudaeginella* spp**. A** *Pseudaeginella montoucheti* (Quitete, 1971). Scale bar 1 mm **B** *Pseudaeginella tristanensis* (Stebbing, 1888) (Redraw from Laubitz 1995) **C** *Pseudaeginella vaderi* Guerra-García, 2004 (Redraw from Guerra-García 2004. Scale bar 1 mm) **D** *Pseudaeginella inae* Krapp-Schickel & Guerra-García, 2005 (Redraw from Krapp-Schickel and Guerra-García 2005. Scale bar 1 mm).

## Supplementary Material

XML Treatment for Pseudaeginella montoucheti
